# Silencing of NRF2 Reduces the Expression of ALDH1A1 and ALDH3A1 and Sensitizes to 5-FU in Pancreatic Cancer Cells

**DOI:** 10.3390/antiox6030052

**Published:** 2017-07-01

**Authors:** Hong-Quan Duong, Kyu Sic You, Seunghoon Oh, Sahng-June Kwak, Yeon-Sun Seong

**Affiliations:** 1 Department of Cancer Research, Vinmec Research Institute of Stem Cell and Gene Technology, 458 Minh Khai, Hanoi 10000, Vietnam; v.quandh1@vinmec.com; 2 Institute of Research and Development, Duy Tan University, K7/25 Quang Trung, Danang 59000, Vietnam; 3 Department of Nanobiomedical Science and BK21 PLUS Global Research Center for Regenerative Medicine, Dankook University, Cheonan 31116, Korea; kisuhezu@gmail.com; 4 Graduate School of Convergence Medical Science, Dankook University, Cheonan 31116, Korea; 5 Department of Physiology, College of Medicine, Dankook University, Cheonan 31116, Korea; seung@dku.edu; 6 Department of Biochemistry, College of Medicine, Dankook University, Cheonan 31116, Korea

**Keywords:** pancreatic cancer, NRF2, ALDH1A1, ALDH3A1, 5-FU

## Abstract

Pancreatic cancer remains an intractable cancer with a poor five-year survival rate, which requires new therapeutic modalities based on the biology of pancreatic oncogenesis. Nuclear factor E2 related factor-2 (NRF2), a key cytoprotective nuclear transcription factor, regulates antioxidant production, reduction, detoxification and drug efflux proteins. It also plays an essential role in cell homeostasis, cell proliferation and resistance to chemotherapy. We aimed to evaluate the possibility that modulation of NRF2 expression could be effective in the treatment of pancreatic cancer cells. We investigated whether the depletion of NRF2 by using small interfering RNAs (siRNAs) is effective in the expression of biomarkers of pancreatic cancer stemness such as aldehyde dehydrogenase 1 family, member A1 (ALDH1A1) and aldehyde dehydrogenase 3 family, member A1 (ALDH3A1). NRF2 knockdown markedly reduced the expression of NRF2 and glutamate-cysteine ligase catalytic subunit (GCLC) in cell lines established from pancreatic cancers. NRF2 silencing also decreased the ALDH1A1 and ALDH3A1 expression. Furthermore, this NRF2 depletion enhanced the antiproliferative effects of the chemotherapeutic agent, 5-fluorouracil (5-FU) in pancreatic cancer cells.

## 1. Introduction

Pancreatic cancer is one of the most lethal solid malignancies and remains the fourth most common cause of cancer-associated mortality worldwide, with a 5-year survival rate of less than 5% [1,2]. The disease is highly aggressive, often discovered late and typically develops resistance to conventional treatment like chemo- and radiotherapy [3]. Therefore, a better understanding of the pathogenic mechanisms involved in the occurrence and progression of pancreatic cancer is required to devise more effective therapeutic strategies.

Cancer stem-like cells have been identified and characterized in various solid tumors, including pancreatic cancer [4,5,6]. Pancreatic cancer stem-like cells are defined as having high tumorigenicity, self-renewal and differentiation capacities [7,8], as well as involvement in chemoresistance along with aggressive behavior [9,10]. The aldehyde dehydrogenase (ALDH) activity is a hallmark of cancer stem-like cells, and thus specific targeting of its isozymes against heterogeneous tumors has implications for anticancer drug development [11,12]. Aldehyde dehydrogenase 1 family, member A1 (ALDH1A1) and aldehyde dehydrogenase 3 family, member A1 (ALDH3A1) have been identified as a biomarker for cancer stem-like cells including pancreatic cancer stem-like cells [11]. They are known to correlate with the resistance to chemotherapeutic agents in pancreatic cancer, hematopoietic stem cells, breast cancer and colon cancer [10,13,14,15]. Furthermore, the expression of ALDH3A1 is robustly higher in DU145-derived prostate cancer stem cells, implying that ALDH3A1 associates with prostate tumorigenesis [16]. Recently, our study showed that combination of dasatinib and gemcitabine exerts anti-proliferative effects by decreasing the expression of ALDH1A1 in pancreatic cancer MIA PaCa-2 cells with acquired resistance to gemcitabine [17].

Nuclear factor E2 related factor-2 (NRF2), a key transcription factor, protects cells against oxidative damage by up-regulating the expression of various genes encoding detoxifying/antioxidant proteins including NAD(P)H quinone oxidoreductase-1 (NQO-1), heme oxygenase-1 (HO-1), glutathione (GSH) generation enzymes and GSH peroxidase (GPx), as well as drug efflux transporters [18,19]. NRF2 dissociates from Kelch-like ECH-associating protein 1 (KEAP1) and accumulates in the nucleus, where it binds to antioxidant response element (ARE) sequence in the regulatory regions of its target genes to induce their expression [20,21,22]. In addition, the constitutive high expression and activity of NRF2 were observed in several tumor types, including pancreatic cancer [23,24]. The elevated NRF2 antioxidant program in cancers occurs through two distinct mechanisms as decreased NRF2 degradation and increased its mRNA transcription due to the activated oncogenic signaling [25]. NRF2 was reported to play a significant role in contribution to chemo- and radioresistance [23,24,26,27]. Moreover, recent studies have indicated the association of NRF2 with cancer stem-like cell function [28,29]. For example, NRF2 knockdown decreases self-renewal capacity of glioma stem cells by significant reduction of the expression of stem-like cell markers as Bmi1, Sox2 and Cyclin E [29].

Along with the findings mentioned above, since pancreatic cancer stem-like cells are poorly differentiated in general and proliferate rapidly, we tried to investigate the contribution of NRF2 to pancreatic oncogenesis by monitoring the expression of ALDH1A1 and ALDH3A1 and measuring the anticancer drug sensitivities under the condition of siRNA-induced NRF2 depletion.

## 2. Materials and Methods

### 2.1. Cell Culture and Reagents

PANC-1 cells were purchased from American Type Culture Collection (ATCC, Manassas, VA, USA). AsPC-1 and COLO-357 cells were obtained from Tissue Culture Shared Resource of Georgetown University Lombardi Comprehensive Cancer Center (Washington, DC, USA) and cultured in Roswell Park Memorial institute (RPMI) 1640 media supplemented with fetal bovine serum (FBS; 20% for AsPC-1 and 10% for Colo-357 cells), 100 units/mL penicillin, 100 µg/mL streptomycin and 1% sodium pyruvate. PANC-1 cells were cultured in Dulbecco’s modified Eagle’s medium (DMEM) containing 10% FBS, 10 units/mL penicillin and 10 µg/mL streptomycin. Cell culture reagents were purchased from BioWhittaker (Walkersville, MD, USA) and Invitrogen (Carlsbad, CA, USA). 5-fluorouracil (5-FU) was purchased from Sigma (St. Louis, MO, USA).

### 2.2. Small Interfering RNA (siRNA) Design and Knockdown Experiment

The siRNA was purchased from Bioneer (Seoul, Korea) with following sequences: NRF2-siRNA, 5′-GAGUAUGAGCUGGAAAAACUU-3′ and control-siRNA, 5′-GACGAGCGGCACGUGCACAUU-3′. NRF2-siRNA or control-siRNA was transfected into pancreatic cancer AsPC-1, COLO-357 and PANC-1 cells using Lipofectamine 2000 (Invitrogen Waltham, MA, USA) as described previously [10]. The siRNA-transfected cells were further analyzed with RT-PCR, western blot and MTT analysis.

### 2.3. RNA Isolation and Reverse Transcription-Polymerase Chain Reaction (RT-PCR)

The RNA isolation and RT-PCR analysis were performed as described previously [23]. Briefly, total RNA from pancreatic cancer cells was isolated with TRIzol Reagent (Invitrogen) after being knocked down by NRF2-siRNA or control-siRNA. Isolated total RNA was then used as the template for reverse transcription using Superscript II reverse transcriptase (Life Technologies Inc., Rockville, MD). The following RT-PCR primers were used: 5′-AAACCACCCTGAAACGACAG-3′ (forward) and AGCGGCTTGAATGTTTGTCT-3′ (reverse) for NRF2; 5′-CTGGGGAGTGATTTCTGCAT-3′ (forward) and 5′-AGGAGGGGGCTTAAATCTCA-3′ (reverse) for GCLC; 5′-TGTTAGCTGATGCCGACTTG-3′ (forward) and 5′-TTCTTAGCCCGCTCAACACT-3′ (reverse) for ALDH1A1; 5′-GCAGACCTGCACAAGAATGA-3′ (forward) and 5′-TGTAGAGCTCGTCCTGCTGA-3′ (reverse) for ALDH3A1; 5′-GCTATCCTGTACGCCTCTG-3′ (forward) and 5′-ACATCTGCTGGAAGGTGGAC-3′ (reverse) for β-Actin. Primer sequences for β-Actin, NRF2, GCLC, ALDH1A1 and ALDH3A1 were obtained from the previous publications [23,30]. Final PCR products were subjected to 2.0% agarose gels electrophoresis containing ethidium bromide along with DNA markers.

### 2.4. Western Blot Analysis

The western blot analysis from pancreatic cancer cells transfected with NRF2-siRNA or control-siRNA was carried out as described previously [31]. Briefly, proteins were separated by sodium dodecyl sulfate-polyacrylamide gel electrophoresis (SDS-PAGE), and transferred to polyvinylidene difluoride membranes (PVDF, Millipore, Bedford, MA, USA). Then, the membranes were blocked in 1X blocking buffer (Sigma, St. Louis, MO, USA) followed by incubation with the following primary antibodies: anti-NRF2 (H-300, Santa Cruz Biotechnology, Santa Cruz, CA, USA), anti-GCLC (ab53179), anti-ALDH1A1 (ab52492) and anti-ALDH3A1 (ab129022) (Abcam, Cambridge, UK) and anti-α-tubulin (T6074, Sigma, St. Louis, MO, USA). After washing three times with washing buffer, the membranes were incubated with horseradish peroxidase (HRP)-conjugated secondary antibodies (Sigma, St. Louis, MO, USA) and the signals were detected by the chemiluminescence kit (Santa Cruz Biotechnology).

### 2.5. 3-(4,5-Dimethylthiazol-2-yl)-2,5-diphenyltetrazolium Bromide (MTT) Assay

Pancreatic cancer cells (2000 cells per well) were plated into 96-well flat bottom plates and treated with two different concentrations (50 μM and 100 μM) of 5-FU for 72 h. After then, 25 µL of 1mg/mL MTT (Sigma, St. Louis, MO, USA) dissolved in PBS was added to each well followed by incubation for 4 h. Finally 150 µL of DMSO (Sigma, St. Louis, MO, USA) was added to each well to dissolve the formazan crystals. The absorbance (560 nm) was measured, and the data was analyzed using an ELx808 Absorbance Microplate Reader (BioTek Instruments, Inc., Winooski, VT, USA). The mean value and standard deviation (SD) were then determined.

### 2.6. Determination of Synergism

For the determination of synergism, classification index (CI) was calculated with the equation of (%A × %B)/(%AB × 100), where %A and %B are the percent cell viability of individual 5-FU treated or NRF2 siRNA transfected cell and %AB is the combination of them. Supra-additivity was defined as CI > 1; additivity was defines as CI = 1; and subadditivity was defined as CI < 1 [32,33].

### 2.7. Statistical Analysis

All data are expressed as mean ± SD of triplicate experiments. Statistical comparison was carried out using two-tailed student’s *t*-test. The results were regarded statistically significant when the *p* value was lower than 0.05. In all experiments, * represents *p* < 0.05, ** represents *p* < 0.01 and *** represents *p* < 0.001.

## 3. Results

### 3.1. NRF2 Knockdown Reduces the Expression of NRF2 and GCLC

**Figure 1:**
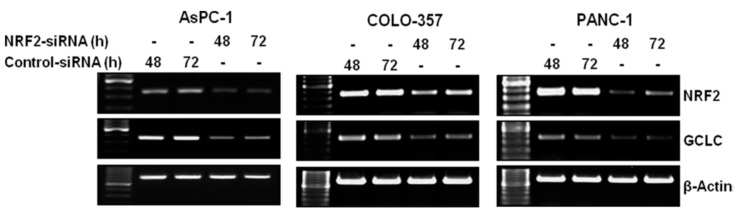
NRF2 knockdown inhibits the mRNA expression of NRF2 and GCLC. AsPC-1, COLO-357 and PANC-1 cells transfected with NRF2-siRNA or control-siRNA for 48 h or 72 h were subjected to RT-PCR analysis using primers specific for NRF2, GCLC and β-Actin.

To evaluate the important role of NRF2 in regulation of the expression of ALDH1A1 and ALDH3A1 in pancreatic cancer cells, we first knocked down the expression of NRF2 in pancreatic cancer AsPC-1, COLO-357 and PANC-1 cells using siRNA. The RT-PCR results at 48 h and 72 h post-transfection revealed that the NRF2 mRNA levels were reduced in NRF2-siRNA transfected AsPC-1, COLO-357 and PANC-1 cells compared with those in control-siRNA transfected cells (Figure 1).

**Figure 2:**
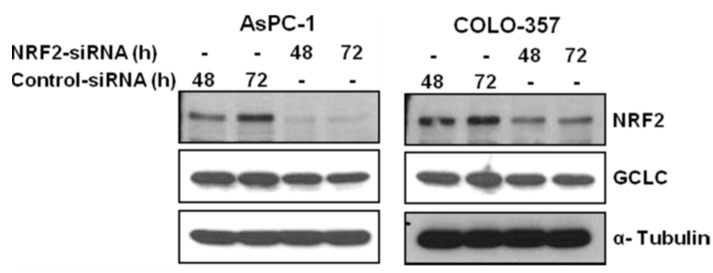
NRF2 knockdown inhibits the protein expression of NRF2 and GCLC. AsPC-1 and COLO-357 cells transfected with NRF2-siRNA or control-siRNA for 48 h and 72 h were subjected to western blot analysis using indicated antibodies. Anti-α-Tubulin antibody was used as a loading and transfer control.

Western blot analysis also showed that the expression of NRF2 was significantly reduced in NRF2-siRNA transfected AsPC-1 and COLO-357 cells, compared to those in control cells (Figure 2).

To further evaluate whether decreased levels of NRF2 downregulated the expression of genes in the NRF2 signaling pathway, we measured the mRNA levels of glutamate-cysteine ligase catalytic subunit (GCLC), one of NRF2 downstream target genes. RT-PCR results showed that the mRNA expression of GCLC were distinctly decreased in NRF2-siRNA transfected AsPC-1, COLO-357 and PANC-1 cells compared to those in control cells (Figure 1). Western blot analysis also showed that the protein expression of GCLC was also significantly decreased in NRF2-siRNA transfected AsPC-1 and COLO-357 cells compared to those in control cells (Figure 2).

### 3.2. NRF2 Knockdown Reduces the Expression of ALDH1A1 and ALDH3A1

**Figure 3:**
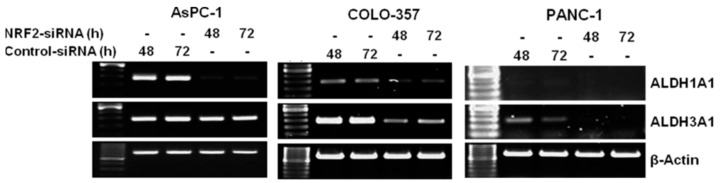
NRF2 knockdown inhibits the mRNA expression of ALDH1A1 and ALDH3A1. AsPC-1, COLO-357 and PANC-1 cells transfected with NRF2-siRNA or control-siRNA for 48 h or 72 h were subjected to RT-PCR analysis using primers specific for ALDH1A1, ALDH3A1 and β-Actin.

To investigate whether silencing NRF2 reduced the expression of ALDH1A1 and ALDH3A1, we performed RT-PCR to determine the effect of NRF2 inhibition by siRNA on ALDH1A1 and ALDH3A1 expression in AsPC-1, COLO-357 and PANC-1 cells. The RT-PCR results demonstrated that the mRNA of ALDH1A1 and ALDH3A1 were significantly decreased in NRF2-siRNA transfected pancreatic cancer cells compared to those in control-siRNA transfected cells (Figure 3).

**Figure 4:**
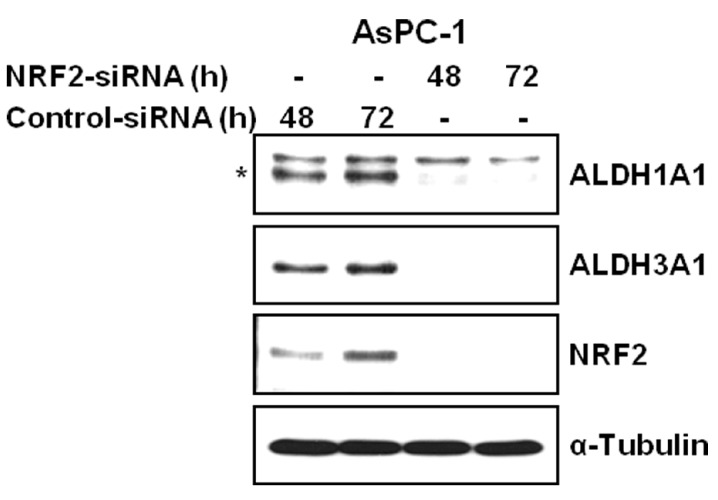
NRF2 knockdown inhibits the protein expression of ALDH1A1 and ALDH3A1. AsPC-1 cells transfected with NRF2-siRNA or control-siRNA for 48 h or 72 h were subjected to western blot analysis using indicated antibodies. Anti-α-Tubulin antibody was used as a loading and transfer control.

Western blot analysis also confirmed the effect of NRF2 knockdown on reducing the expression of ALDH1A1 and ALDH3A1. Compared to cells transfected with control-siRNA, cells transfected with NRF2-siRNA showed the distinct decrease in the protein expression of ALDH1A1 and ALDH3A1 in AsPC-1 cells (Figure 4), suggesting that NRF2 knockdown suppresses the expression of ALDH1A1 and ALDH3A1. The NRF2 dependent expression of ALDH1A1 and ALDH3A1 was in accord with our previous experiment of cDNA microarray study after knockdown or induction of NRF2 in AsPC-1 cell line [34].

**Figure 5:**
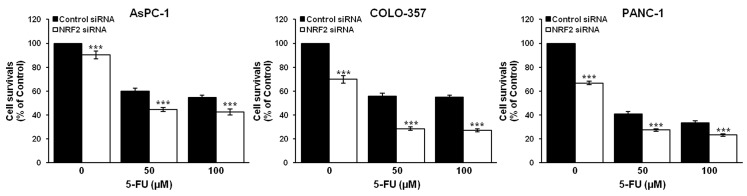
NRF2 knockdown sensitizes pancreatic cancer cells AsPC-1, COLO-357 and PANC-1 to chemotherapeutic agent 5-FU. AsPC-1, COLO-357 and PANC-1 cells were transfected with NRF2-siRNA or control-siRNA for 48 h and further treated with 5-FU at different concentrations (0, 50 and 100 µM) for 72 h. They were then subjected to cell viability assay using MTT. Error bars represent standard deviation. *** *p* < 0.001 represents the significant difference between NRF2 siRNA plus 5-FU group (filled bar) and control siRNA plus 5-FU group (open bar).

Since the highly expressed NRF2 levels potentiated the resistance to chemotherapeutic agents in pancreatic cancer cells, we then investigated the role of NRF2 in determination of the sensitivity of AsPC-1, COLO-357 and PANC-1 cells to the chemotherapeutic agents 5-fluorouracil (5-FU). NRF2-depleted or control cells at 48 h post-transfection were treated with two different concentrations of 5-FU (0, 50 or 100 µM) for 72 h. The results of MTT assay revealed that the depletion of NRF2 by siRNA significantly enhanced the sensitivity of pancreatic cancer cells to 5-FU (Figure 5). Due to the limited numbers of combination the calculation of classification index (CI) was chosen to assess synergistic effect of combination rather than combination index [32,33]. The calculated CI of 5-FU and NRF2 knockdown combination revealed that AsPC-1 and COLO-357 cell lines showed supra-additivity with mean CI values 1.35, 1.23 (50 μM, 100 μM 5-FU each in AsPC-1 cell line); 1.49, 1.55 (50 μM, 100 μM 5-FU each in COLO-357 cell line). However, in PANC-1 cells the combination effect was minimally supra-additive with mean CI values 1.06, 1.03 (50 μM, 100 μM 5-FU each).

All together, these results suggest that inhibition of NRF2 is related with the enhancement of the sensitivity of these cells to chemotherapeutic agents and the expression of cancer cell stemness biomarker as ALDH1A1 and ALDH3A1.

## 4. Discussion

Over the last decade, numerous studies have shaped the hierarchical theory to explain the intra-tumoral heterogeneity observed in many tumors including pancreatic cancer [4,7,8,9]. This theory argues that a tumor comprises cells of not just a single clone, but multiple subclones with distinct cell morphologies, gene expression profiles, cell growth kinetics and cell surface markers. Among these heterogeneous subclones, a minor one generally comprising less than 5% of a tumor is termed the cancer stem cell. It has a potential for unlimited self-renewal with the capacity to provide committed progeny constituting the volume of a tumor through asymmetric cell division. This feature naturally renders cells of this specific subclone responsible for cancer initiation, progression, invasion, metastasis and resistance to various modes of therapy [7,8,9,10]. In pancreatic cancer, investigation of cancer stem cell lineages enabled the identification of cell membrane markers such as CD44, CD24, epithelial specific antigen (ESA) and CD133 and the expression pattern of ALDH1A1 as an important biomarker of pancreatic cancer stem cells [8,35]. Considering the poor outcomes from conventional treatments currently available for pancreatic cancer, more effective therapy needs immediate development, and pancreatic cancer stem cells can be a highly desirable target worthy of investigation. Related to this study, our recent study has demonstrated that dasatinib enhances gemcitabine-induced decreased ALDH1A1 expression and increased cell death of MIA PaCa-2 pancreatic cancer cells with acquired resistance to gemcitabine [17].

The biochemical role of NRF2 and its representative downstream proteins are: (1) NADPH production for reduction and monooxygenation: glucose-6 phosphate dehydrogenase (G6PD), a rate-limiting step enzyme of NADPH production, 6-phosphogluconate dehydrogenase (6PGD) and malic enzyme (ME); (2) glutathione dependent superoxide removal system: GCLC, several kinds of superoxide dismutases (SODs), glutathione reductase (GSR) and the family of thioredoxin reductases (TXNRDs); (3) drug conjugating or oxidizing system: several kinds of glutathione *s*-transferases (GSTs), cytochrome p450 family enzymes (CYPs), NQO-1 an oxidized mitochondrial quinone detoxifying enzyme, aldo-keto reductase family enzymes (AKRs) and ALDHs; and (4) drug efflux system: the family of multidrug resistant proteins (MDRs) and ABC transporters [19,36,37]. In addition to catalyzing the endogenous substrate, the substrate cross-reactivities of these proteins enable them to turn over xenobiotics to eliminate them from the body, or to produce metabolic products with unidentified function.

NRF2 protects cells from oxidative stress by upregulating the expression of various kinds of genes involved in cytoprotection and detoxification [18,19]. The protective role of NRF2 from oxidants and electrophiles is essential in various malignant cancers, where high expression of NRF2 can promote cancer cell viability, growth and resistance to conventional chemotherapy and radiotherapy [23,24,26,27]. NRF2 has also been shown to be an essential factor of differentiation and self-renewal capability of glioma stem cells [28]. Silencing NRF2 decreases the self-renewal capacity of glioma stem cells by critically reducing the expression of B lymphoma Mo-MLV insertion region 1 homolog (Bmi1, a transcription regulation factor for stem cells), SRY-box 2 (Sox2, a regulator of growth factors) and Cyclin E [28]. NRF2 also regulates hematopoietic stem cell survival [38,39]. In contrast, inhibition of NRF2-dependent antioxidant response by siRNA in various cancers, including pancreatic cancer, can lead to increased sensitivity to gemcitabine, 5-FU, cisplatin, camptothecin, etoposide and doxorubicin [23,24,31]. Consistent with previous studies, this study also revealed that NRF2 inhibition by siRNA enhanced the sensitivity of pancreatic cancer AsPC-1, COLO-357 and PANC-1 cells to 5-FU. These observations support that decreasing NRF2-dependent protective response can promote the sensitivity of cancer cells including pancreatic cancer to anticancer therapeutic agents. The degradation of 5-FU is initiated by the reduction to dihydrofluorouracil by dihydropyrimidine dehydrogenase, a rate-liming NADPH dependent enzyme [40]. The 5-FU treatment increases intracellular reactive oxygen species (ROS) and the removal of them by antioxidants rescues cancer cells from apoptotic death [41]. We also previously reported that the synergistic effect of chk2 inhibitor with gemcitabine revealed increased production of ROS and the ROS scavenger treatment reversed apoptotic cell death [42]. This protective effect of NRF2 arises from the effectiveness of removal of ROS under the treatment of cytotoxic drugs and possibly the increased viability of cells due to the NRF2 related stemness property [43].

Although the mechanism or direct temporal cause-result relationship between chemoresistance and the expression of ALDH1A1 and ALDH3A1 was not addressed in this experiment, the role of NRF2 in pancreatic cancer cell survival is evident by modulating the expression of these drug metabolizing enzymes including possibly ALDH1A1, ALDH3A1 and other potent downstream target of NRF2 [37,44]. The pharmacological inhibition of ALDH1A1 resulted in decreased formation of sphere-like colonies along with down regulation of cancer stem cell markers in head and neck squamous cell carcinoma cell lines [45]. In addition to ALDH1A1 and ALDH3A1, ALDH7A1 is also known as an ALDH family member of NRF2 downstream targets. ALDH7A1 is functionally involved with stemness and metastatic activity of prostate cancer and recurrence of non-small cell lung cancer [46,47]. Beyond to the simple enzymatic function of detoxification as aldehyde turn over to carboxylic acid, ALDHs have numerous non-enzymatic functions. The production of retinoic acids, γ-amino butyric acid and other unidentified carboxylic acid products may participate various cellular processes including cell proliferation, differentiation and survival [12]. Interestingly, in our previous study, siRNA-mediated ALDH1A1 knockdown inhibited cell proliferation in pancreatic cancer MIA PaCa-2 cells [10]. Moreover, the treatment of ALDH1A1-siRNA perturbed gemcitabine resistance, resulting in decreased cell viability, increased apoptotic cell death and the accumulation of cells at S-phase [10].

The effective modulation of specific NRF2 downstream effectors could be a therapeutic target for the development of novel therapy for pancreatic cancer. Further detailed study, such as the functional assay of sphere formation under the knockdown of ALDH1A1 or ALDH3A1, is needed to define the molecular mechanisms involved in the function of NRF2 and its effectors, including members of ALDHs in pancreatic cancer cells in relevance with drug resistance and cancer cell stemness.
